# BMI category-specific waist circumference thresholds based on cardiovascular disease outcomes and all-cause mortality: Tehran lipid and glucose study (TLGS)

**DOI:** 10.1186/s12889-023-16190-w

**Published:** 2023-07-05

**Authors:** Amirhosein Seyedhoseinpour, Maryam Barzin, Maryam Mahdavi, Majid Valizadeh, Fereidoun Azizi, Sahar Ghareh, Farhad Hosseinpanah

**Affiliations:** 1 Obesity Research Center, Research Institute for Endocrine Sciences, Shahid Beheshti University of Medical Sciences, Tehran, Iran; 2 Endocrine Research Center, Research Institute for Endocrine Sciences, Shahid Beheshti University of Medical Sciences, Tehran, Iran; 3 Tehran Branch, Faculty of Medicine, Islamic Azad University, Tehran, Iran

## Abstract

**Background:**

Waist circumference (WC), a representative of abdominal visceral fat, is strongly associated with cardiovascular disease (CVD) and its outcomes. We aimed to define body mass index (BMI)-specific WC thresholds as predictors of CVD and all-cause mortality.

**Methods:**

In this prospective cohort study in the context of the Tehran Lipid and Glucose Study (TLGS), a total of 3344 men and 4068 women were followed up for 18 years. Based on BMI, the participants were categorized into three groups: BMI < 25, 25 < BMI < 30, and BMI > 30. In each BMI category, sex-specific WC thresholds were estimated by the maximum value of Youden’s index to predict based on incident CVD events and all-cause mortality prediction.

**Results:**

Overall 667 and 463 CVD events (the incidence rate of 3.1 to 4.5 in men and 1.1 to 2.6 in women per 1,000 person-years within BMI categories) and 438 and 302 mortalities (the incidence rate of 2.1 to 2.7 in men and 1.2 to 1.4 in women per 1,000 person-years within BMI categories) were recorded in men and women, respectively. WC thresholds in the BMI categories of < 25, 25–30, and BMI > 30 kg/m^2^ with regard to CVD events were 82, 95, and 103 cm in men and 82, 89, and 100 cm in women, and regarding all-cause mortality, the respective values were 88, 95, and 103 cm in men and 83, 90, and 99 cm among women.

**Conclusion:**

BMI-specific WC thresholds observed here can help to better identify individuals at high risk of developing CVDs.

**Supplementary Information:**

The online version contains supplementary material available at 10.1186/s12889-023-16190-w.

## Introduction

Obesity is a global public health issue [1] and a modifiable major risk factor for cardiovascular disease (CVD) [2]. The prevalence of obesity has increased remarkably in recent years in developing and developed countries [3]. A 33-year long evaluation up until 2013 certified the failure of nationwide preventive measures against obesity in almost all countries around the world [4]. The trend of obesity in Iran revealed an increasing prevalence from 13.6 to 22.3% from 1999 to 2007 [5]. Also, the evidence provided by the Tehran Lipid and Glucose Study (TLGS), a 12- year longitudinal study (from 1999 to 2011) in Tehran, Iran’s capital city, showed that the prevalence of obesity and abdominal obesity increased from 23.1% to 47.9% at the baseline to 34.1% and 71.1% at the end of follow-up, respectively [6].

Waist circumference (WC) is an easily measurable anthropometric parameter. Unlike the body mass index (BMI), which only represents total body fat mass, WC is a reliable predictor of not only total body fat but also abdominal visceral fat [7]. Compared to total body fat, abdominal visceral fat has more strongly been associated with BMI-adjusted CVD incidence [8]. Also, WC strongly correlates with the incidence of metabolic syndrome [9], all-cause mortality, and the development of CVD [10–12] In fact, WC is considered an independent risk factor for CVD and CVD-related mortality [13], showing an even stronger predictive value after adjustment for BMI [12–14].

Regarding different BMI categories, health problems increase from the normal weight towards overweight and obesity groups. Also, in individual BMI categories, people with higher WC represent a higher risk for CVD and mortality [15, 16]. A wide range of WC cut-off points have been defined utilizing different methodologies; however, these WC thresholds vary in different populations and ethnic groups, so the same thresholds cannot be applicable to all populations [17]. In the current guidelines, the most commonly used WC cut-off point for white Caucasians is ≥ 102 cm for men and ≥ 88 cm for women, presenting a sex-specific equivalent to BMI = 30 kg/m^2^ [18]. Several studies have suggested different sex-specific cut-off values for WC to predict the risk of developing cardio metabolic disorders [19–21], and CVD events [22, 23]. However, many of these studies are cross-sectional studies and, therefore, cannot provide conclusive evidence BMI-specific WC cut-off points have been associated with better sensitivity and specificity for predicting cardio metabolic events [24] and mortality compared to sex-specific thresholds [25].

In this prospective cohort study, which was conducted in the framework of the Tehran Lipid and Glucose Study (TLGS), we aimed to evaluate the predictive value of BMI-specific WC thresholds for CVD events and all-cause mortality in order to identify at-risk the patients referring to health care clinics and to offer an effective screening measure at the community scales.

## Methods and materials

### Study population

In this prospective cohort study, the subjects were recruited from the TLGS, an ongoing community-based prospective study to determine the risk factors and outcomes of non-communicable diseases [26]. In phases I and II of TLGS, 15,005 participants were recruited through the multistage random sampling method from District 13 of Tehran. In the framework of TLGS, the demographic, clinical, biochemical, anthropometric, and lifestyle data of the participants were gathered every three years from 1999 to 2001. Further prospective follow-ups were held from 2002 to 2005 (phase II), 2006 to 2008 (phase III), 2009 to 2011 (phase IV), 2012 to 2015 (phase V), and 2016 to 2019 (phase VI).

Out of 9559 participants, aged ≥ 30 years, enrolled in phases I and II of the study, we excluded a number of them due to having a history of CVD at the baseline (n = 785), history of cancer (n = 43), consuming corticosteroids at the baseline (n = 180), being underweight (n = 112), or missing data or lost to follow-up (n = 1027). Finally, 7412 participants were enrolled in the present study, of whom 3344 were men, and 4068 were women. All the participants were followed up until 20 March 2018 with a median follow-up duration of 17.7 years.

### Ethical approval

This study was conducted according to the guidelines of the Declaration of Helsinki, and all its procedures involving human subjects were approved by the research committee of Shahid.

Beheshti University of Medical Sciences. Written informed consent was obtained from all subjects.

### Anthropometric measurements

The detailed protocols and laboratory procedures of the TLGS have been published elsewhere [26]. Briefly, all demographic and anthropometric data were collected by trained health care professionals, using established protocols. While the subjects were standing barefoot against a wall with shoulders in the normal alignment, a tape stadiometer was used to measure height. Weight was measured using a digital electronic scale (Seca 707; range 0.1–150 kg, Hanover, MD, USA) while the subjects were minimally clothed and barefoot, and the obtained weight was rounded to the nearest 100 g. Body mass index was measured as $Weight\left(kg\right)/Height\left(m\right)^2$. Waist circumference was measured using a non-flexible tape meter at the umbilical level without pressuring the body surface while the subjects were standing, and the measurements were rounded to the nearest 0.1 cm. After the subjects rested for 15 min, systolic and diastolic blood pressures (SBP and DBP) on the right brachial artery at the heart level were recorded at least twice by a physician utilizing a standard mercury sphygmomanometer calibrated by the Iranian Institute of Standards and Industrial Researches.

### Laboratory assessments

After 12 to 14 h of fasting, blood samples were collected from 7 to 9 am at the TLGS Research Laboratory to measure fasting plasma glucose (FPG) and lipid profile parameters utilizing commercially available laboratory kits (Pars Azmoon Inc, Tehran, Iran) adapted to a Selectra 2 auto-analyzer. The blood samples were centrifuged for 30–45 min after collection. An enzymatic calorimetric method with glucose oxidase was used for FPG; glycerol phosphate oxidase for triglyceride (TG); and cholesterol esterase and cholesterol oxidase for total cholesterol (TC). High-density lipoprotein cholesterol (HDL-C) was measured after precipitating apolipoprotein B-containing lipoproteins with phosphotungstic acid. If TG was obtained < 400 mg/dl, the level of low-density lipoprotein cholesterol (LDL-C) was calculated using the Friedwald formula [27]. The inter and intra-assay coefficients of variation (CV) were 0.6 and 1.6% for TG; 0.5 and 2% for HDL-C; 2.2 and 2.2% for FPG; and 0.5 and 2% for TC, respectively [26].

### Definitions

A family history of CVD was defined as a prior diagnosis of CVD in any first-degree women relative aged < 65 years or a men relative aged < 55 years old. Hypertension is diagnosed as SBP ≥ 140 mmHg and/or DBP ≥ 90 mmHg, or the current use of antihypertensive medication [28]. Diabetes mellitus is defined as the presence of each of the following criteria; 1- Fasting plasma glucose level ≥ 7.0 mmol/L (126 mg/dL), 2- Plasma glucose ≥ 11.1 mmol/L (200 mg/dL) two hours after consuming 75-gram oral glucose, 3- Plasma glucose ≥ 11.1 mmol/L (200 mg/dL) in the presence of hyperglycemia symptoms, 4- The current use of diabetes medication [29].

Coronary heart disease (CHD) is defined as 1- definite myocardial infarction (MI); diagnosed by evolving diagnostic electrocardiography (ECG) and positive biomarkers; or, 2- probable myocardial infarction; diagnosed by positive ECG findings plus cardiac symptoms and signs plus missing biomarkers, or Positive ECG findings plus equivocal biomarkers; or, 3- unstable angina pectoris; defined as new cardiac symptoms or changing symptom patterns and positive ECG findings and normal biomarkers; or 4- angiographic proven CHD; or 5- CHD death; including definite and possible fatal MI. CVD is defined as the presence of CHD as defined priorly, or stroke; or cerebrovascular death [30]. Cardiovascular mortality is defined as death attributed to MI, heart failure, cardiac arrest due to other or unknown causes, or cerebrovascular accidents [31].

### Statistical analysis

All statistical analyses were stratified by sex. The normality of data distribution was assessed by visually inspecting data histograms and based on the output of the Shapiro-Wilk test. All normally-distributed continuous variables were expressed as mean ± standard deviation (SD) and continuous variables with skewed distributions were described by median and the inter-quartile range (IQR) 25–75. Categorical variables of baseline characteristics were shown as frequency (percentages). Gender-stratified differences in participants’ characteristics in characteristics between different BMI categories were tested using analysis of variance (ANOVA), Kruskal-Wallis H, and the Chi-square test for normally distributed continuous variables, skewed, continuous variables and categorical variables, respectively. Cox proportional hazard models were used to estimate the hazard ratios (HRs) with 95% confidence intervals (95% CIs) for CVD events and all-cause mortality in BMI categories. The Akaike information criterion (AIC) and Bayesian information criterion (BIC) were also calculated in each model. Cut-off points for WC in different BMI categories were estimated by the maximum value of Youden’s index. The discriminatory powers of waist circumference within each BMI categories in univariate models for CVD events and all-cause mortality, calculated by the C index. We assessed discrimination based on Harrell’s concordance statistic (c-index) and differences between c-index was assessed using lincom in STATA. All statistical analyses were performed in STATA version 14 (STATA, College Station, TX); the statistical significance level was set at P *<* 0.05 (two-tailed).

## Results

Among all the subjects recruited in phases I and II of TLGS, after excluding non-eligible subjects, 7412 individuals aged above 30 years old were enrolled, of whom 3344 were men (45.1%) and 4068 were women (54.9%). The baseline characteristics and cardio-metabolic profiles of men and women participants are presented in Tables 1 and 2, respectively. The mean age was 47.2±12.9 years in men and 45.8±11.4 years in women. Of all men, 1264 (37.8%) were normal weight, 1541 (46.1%) were overweight, and 539 (16.1%) were obese. Among women, 919 (22.6%), 1708 (42.0%), and 1441 (35.4%) were in the normal weight, overweight, and obese categories, respectively. The number of normal weight individuals was lower among women compared to men, and more women were categorized under the obese category than men. The mean of WC was 91.1±10.5 cm in men and 90.7±11.9 cm in women. Regarding the BMI categories of < 25, 25–30, and > 30 kg/m^2^, 81.8±6.7, 93.8±6.1, and 105±7.7 cm in men and 78.2±7.3, 88.7±7.8 and 100.9±9.0 cm in women, respectively, showing significant differences between the categories in both sexes. Gender-stratified comparison of the participants’ characteristics between different BMI categories revealed significant differences in all variables in both men and women, except for the family history of CVD and smoking in women. From the BMI categories of < 25 to 25–30 and then > 30 kg/m^2^, the mean values of cardio metabolic parameters showed significant increasing trends in both men and women, including SBP (116±17 to 123±18 to 127±20 in men; and 114±18 to 120±20 to 126±20 mmHg in women), DBP (75±11 to 80±11 to 83±11 in men; and 74±10 to 78±10 to 83±11 mmHg in women), FPG (95±28 to 100±30 to 105±33 in men; and 95±33 to 101±37 to 104±36 mg/dL in women), 2-h plasma glucose concentration (104±47 to 123±65 to 132±65 in men; and 110±44 to 125±56 to 135±58 mg/dL in women), TC (197±40 to 211±43 to 216±40 in men; and 203±43 to 220±47 to 226±47 mg/dL in women), LDL-cholesterol (128±35 to 134±36 to 138±36 in men; and 130±37 to 141±39 to 144±39 mg/dL in women) and TG (129 to 179 to 203 in men; and 109 to 151 to 177 mg/dL in women). Also, a significant decreasing trend was observed in HDL-cholesterol in both sexes (39±10 to 37±9 to 36±9 in men; and 47±11 to 44±11 to 43±10 mg/dL in women). A total of 1872 subjects had a positive history of diabetes mellitus, of whom 1129 were men and 743 were women. The percentage of diabetic subjects within BMI categories significantly increased in both sexes from BMI < 25 (15.1% in men and 18.0% in women) to 25 > BMI < 30 (26.0% in men and 27.7% in women), and then BMI > 30 kg/m^2^ (35.4% in men and 37.2% in women) the same trend was also observed for the ratio of e subjects with hypertension (14.3–24.8 to 32.8% in men; and 14.5–22.9 to 37.7% in women, respectively).

**Table 1:** Baseline characteristics of **Men** participants

	BMI < 25 N = 1264 (37.8%)	25 < BMI < 30 N = 1541 (46.1%)	BMI > 30 N = 536 (16.1%)	Total N = 3344	*P*-Value
**Age** (year)	47.4 ± 13.7	47.2 ± 12.4	46.8 ± 12.3	47.2 ± 12.9	0.559
**Weight** (kg)	65.0 ± 6.9	78.2 ± 7.2	92.5 ± 10.1	75.5 ± 12.2	< 0.001
**BMI** (kg/m^2^)	22.6 ± 1.7	27.2 ± 1.4	32.4 ± 2.6	26.3 ± 3.8	< 0.001
**WC** (cm)	81.8 ±6.7	93.8 ± 6.1	105.0 ± 7.7	91.1 ± 10.5	< 0.001
**Family history of CVD**, n(%)	146 (11.6)	211 (13.7)	102 (19.0)	459 (13.8)	< 0.001
**Smoking**, n(%)	435 (34.6)	495 (25.7)	129 (24.0)	959 (28.8)	< 0.001
**SBP** (mmHg)	116.4 ± 17.2	122.8 ± 18.2	127.3 ± 19.8	121.1 ± 18.6	< 0.001
**DBP** (mmHg)	74.8 ± 10.5	79.7 ± 10.8	82.9 ± 10.9	78.4 ±11.1	< 0.001
**HTN**, n(%)	178 (14.3)	379 (24.8)	173 (32.4)	730 (22.1)	< 0.001
**FPS** (mg/dL)	95.4 ±27.5	100.2 ± 30.1	105.2 ± 33.4	99.2 ± 29.9	< 0.001
**2hBG**(mg/dL)	103.8 ± 46.6	123.4 ± 64.9	131.8 ± 65.3	117.4 ± 59.8	< 0.001
**DM**, n(%)	180 (15.1)	379 (26.0)	184 (35.4)	743 (23.5)	< 0.001
**TC** (mg/dL)	197.1 ± 40.4	210.7 ± 42.7	215.8 ± 40.1	206.4 ± 42.1	< 0.001
**HDL-C** (mg/dL)	39.4 ± 9.9	36.6 ± 8.8	36.1 ± 8.9	36.1 ±8.9	< 0.001
**LDL-C** (mg/dL)	127.6 ± 34.7	134.1 ± 36.2	137.9 ±35.6	132.2 ± 35.8	< 0.001
**TG** (mg/dL) **‡**	129.0 (89.8–183.0)	178.5 (130.0-253.8)	202.5 (149.0-267.8)	163.0 (113.0-233.0)	< 0.001

BMI, body mass index; 2hPG, 2 h post-prandial blood sugar; TC, total cholesterol; CVD, cardiovascular disease; DBP, diastolic blood pressure; DM, diabetes mellitus; HDL-C, high-density lipoprotein cholesterol; HTN, hypertension; LDL-C, low-density lipoprotein cholesterol; SBP, systolic blood pressure; TG, triglyceride.

Data are given as the mean (SD) or median (IQ 25–75) unless otherwise indicated (‡).

**Table 2:** Baseline characteristics of **Women** participants

	BMI < 25 N = 919 (22.6%)	25 < BMI < 30 N = 1708 (42.0%)	BMI > 30 N = 1441 (35.4%)	Total N = 4068	*P*-Value
**Age** (year)	43.2 ± 12.3	46.0 ± 11.5	47.3 ± 10.6	45.8 ± 11.4	< 0.001
**Weight** (kg)	56.2 ± 5.8	66.9 ± 5.7	80.6 ± 9.8	69.4 ± 11.9	< 0.001
**BMI** (kg/m^2^)	22.9 ± 1.6	27.5 ± 1.4	33.5 ± 3.2	28.6 ± 4.6	< 0.001
**WC** (cm)	78.2 ± 7.3	88.7 ± 7.8	100.9 ± 9.0	90.7 ± 11.9	< 0.001
**Family history of CVD**, n (%)	151 (16.5)	301 (17.7)	276 (19.2)	728 (17.9)	0.222
**Smoking**, n (%)	39 (4.3)	64 (3.8)	51 (3.5)	154 (3.8)	0.670
**SBP** (mmHg)	113.7 ± 17.9	120.3 ± 19.6	126.3 ± 20.2	120.9 ± 20.0	< 0.001
**DBP** (mmHg)	73.9 ± 9.8	78.1 ± 10.0	82.6 ± 10.8	78.8 ± 10.8	< 0.001
**HTN**, n (%)	132 (14.5)	390 (22.9)	539 (37.7)	1061 (26.2)	< 0.001
**FPS** (mg/dL)	95.3 ± 33.5	100.6 ± 36.6	104.2 ± 36.4	100.7 ± 36.0	< 0.001
**2hBG**(mg/dL)	109.9 ± 43.8	125.1 ± 56.2	135.3 ± 58.0	125.2 ± 55.1	< 0.001
**DM**, n (%)	157 (18.0)	456 (27.7)	516 (37.2)	1129 (28.9)	< 0.001
**TC** (mg/dL)	203.4 ± 42.9	219.7 ± 47.1	226.0 ± 47.1	218.3 ± 46.9	< 0.001
**HDL-C** (mg/dL)	46.9 ± 11.0	43.9 ± 11.3	43.0 ± 10.3	44.3 ± 11.0	< 0.001
**LDL-C** (mg/dL)	130.4 ± 36.6	141.4 ± 39.0	144.1 ± 38.9	139.8 ± 38.8	< 0.001
**TG** (mg/dL) **‡**	109.0 (80.0-158.0)	151.0 (105.0-213.0)	177.0 (127.0-235.0)	150.0 (103.0-212.0)	< 0.001

BMI, body mass index; 2hPG, 2 h post-prandial blood sugar; TC, total cholesterol; CVD, cardiovascular disease; DBP, diastolic blood pressure; DM, diabetes mellitus; HDL-C, high-density lipoprotein cholesterol; HTN, hypertension; LDL-C, low-density lipoprotein cholesterol; SBP, systolic blood pressure; TG, triglyceride.

Data are given as the mean (SD) or median (IQ 25–75) unless otherwise indicated (‡).

The incidence rates and hazard ratios (per 1 cm increase in WC within each BMI category) of incident CVD and all-cause mortality were depicted in Table 3. During the follow-up, 667 (19.9%) men and 463 (11.4%) women were diagnosed with CVD. There were 740 deaths, of which 438 occurred among men and 302 among women. Overall, 32% (140 out of 438) and 24.2% (73 out of 302) of all deaths in men and women were due to CVD, respectively. The rate of CVD events showed an increasing trend from the normal weight to obese categories in both men (0.31, 0.41, and 0.45 per 10,000 person years, respectively) and women (0.11, 0.21, and 0.26 per 10,000 person years, respectively). Also, a higher CVD incidence rate was observed among men than women across all BMI categories. The rates of all-cause mortality per 10,000 person-years were 0.27 (normal weight), 0.21 (overweight), and 0.22 (obese) in men and 0.12 (normal weight), 0.11 (overweight), and 0.14 (obese) in women, showing the highest and lowest all-cause mortality rates in men with a BMI of < 25 kg/m^2^ (0.27 per 10,000 person-years), and women with 25 < BMI < 30 (0.14 per 10,000 person-years). Overall, all-cause mortality was higher in men compared to women across all BMI categories.

**Table 3:** Incident cardiovascular disease (CVD) and all-cause mortality in BMI categories and hazard ratios per 1 cm increase in WC within each BMI categories

	Men			Women		
	BMI < 25	25 < BMI < 30	BMI > 30	BMI < 25	25 < BMI < 30	BMI > 30
CVD						
No of person-year	6,626,515	8,106,047	2,808,150	5,301,946	9,521,589	7,897,815
No of CVD incident	206	335	126	60	196	207
Incidence rate (Per 10,000 person-years)	0.31 (0.27 − 0.36)	0.41 (0.37 − 0.46)	0.45 (0.38 − 0.54)	0.11 (0.09 − 0.15)	0.21 (0.18 − 0.24)	0.26 (0.23 − 0.30)
Hazard ratios (95%CI) (Per 1 cm increase in WC)	1.054 (1.03 − 1.07)	1.041 (1.02 − 1.06)	1.035 (1.01 − 1.05)	1.081 (1.05 − 1.11)	1.068 (1.05 − 1.09)	1.041 (1.03 − 1.06)
AIC	2806.24	4725.48	1504.06	772.468	2786.81	2870.84
BIC	2811.38	4730.82	1508.35	777.291	2792.25	2876.11
**All-cause mortality**						
No of person-year	6,968,967	8,826,047	3,054,811	5,403,171	9,937,393	8,307,184
No of CVD incident	189	183	66	66	117	119
Incidence rate (Per 10,000 person-years)	0.27 (0.23 − 0.31)	0.21 (0.18 − 0.24)	0.22 (0.17 − 0.28)	0.12 (0.09 − 0.15)	0.11 (0.09 − 0.14)	0.14 (0.12 − 0.17)
Hazard ratios (95%CI) (Per 1 cm increase in WC)	1.047 (1.02 − 1.07)	1.034 (1.01 − 1.06)	1.029 (1.00 − 1.06)	1.056 (1.02 − 1.09)	1.123 (1.10 − 1.15)	1.068 (1.05 − 1.09)
AIC	2596.94	2604.76	799.27	865.34	1593.89	1609.77
BIC	2602.08	2610.10	803.55	870.16	1599.33	1615.04

BMI, body mass index; CI, confidence interval; CVD, cardiovascular disease; WC, waist circumference

BMI-specific WC thresholds based on CVD events and all-cause mortality have been demonstrated in Table 4. The WC cut-off point predicting CVD events in normal weight men (BMI < 25) was 82 cm, while a higher cut-off point (88 cm) was obtained for all-cause mortality. The WC cut-off points predicting CVD events and all-cause mortality were similarly 95 cm in 25 > BMI > 30 and 103 cm in BMI > 30. Among women, the WC thresholds predicting CVD events and all-cause mortality were almost the same in the normal weight and overweight groups (82 and 83 cm in BMI < 25 groups; 89 and 90 cm in 25 > BMI > 30; and 100 and 99 in BMI > 30, respectively). The sensitivity and specificity of the predictive WC cut-off points for CVD events and all-cause mortality were presented in Supplementary Tables 1 and 2, respectively.

**Table 4:** Waist circumference cut-points predicting cardiovascular disease and all-cause mortality

	Sex	BMI Category	Cut-point	Sensitivity	Specificity	Youden’s index	C-Index (95% CI)
**CVD**	**Men**	**BMI < 25**	82	0.709	0.454	0.163	0.601 (0.56 − 0.64)
		**25 < BMI < 30**	95	0.607	0.521	0.128	0.573 (0.54 − 0.60)
		**BMI > 30**	103	0.749	0.382	0.131	0.572 (0.52 − 0.62)
	**Women**	**BMI < 25**	82	0.577	0.681	0.258	0.677 (0.60 − 0.75)
		**25 < BMI < 30**	89	0.791	0.476	0.267	0.653 (0.62 − 0.69)
		**BMI > 30**	100	0.756	0.440	0.196	0.613 (0.57 − 0.65)
**All-cause Mortality**	**Men**	**BMI < 25**	88	0.317	0.761	0.078	0.599 (0.56−-0.64)
		**25 < BMI < 30**	95	0.534	0.540	0.074	0.573 (0.54 − 0.60)
		**BMI > 30**	103	0.780	0.391	0.171	0.569 (0.52 − 0.62)
	**Women**	**BMI < 25**	83	0.551	0.710	0.261	0.677 (0.60 − 0.75)
		**25 < BMI < 30**	90	0.863	0.532	0.386	0.653 (0.62 − 0.69)
		**BMI > 30**	99	0.932	0.407	0.339	0.613 (0.57 − 0.65)

CVD; Cardio –vascular disease, BMI; body mass index.

**Fig. 1:**
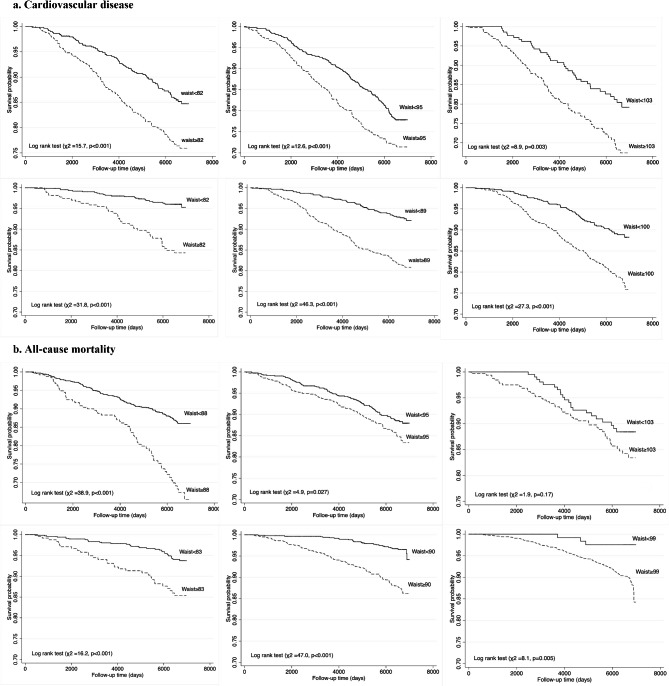
Kaplan-Meier survival curves examining waist circumference thresholds based on incident cardiovascular disease (**a**) all-cause mortality (**b**) among BMI categories

According to Kaplan-Meier survival curve analysis, comparing the probability of survival/incident below and above the specified cut-off points for CVD events and all-cause mortality (Fig. 1), the C-index ranged from 0.569 to 0.677 indicating no significant difference between the thresholds for CVD events and all-cause mortality. Although, the C-index was slightly higher in women than in men, the difference was statistically significant only in the overweight group (Table 4). The same results were obtained after sensitivity analysis and exclusion of the incidents happening in the first two years of the follow-up.

## Discussion

This large prospective cohort study intended to determine BMI-specific WC thresholds for predicting CVD events and all-cause mortality. According to our results, the WC thresholds in the BMI categories of < 25, 25–30, and BMI > 30 kg/m^2^ with regard to CVD events were 82, 95, and 103 cm in men and 82, 89, and 100 cm in women, and regarding all-cause mortality, the respective values were 88, 95, and 103 cm in men and 83, 90, and 99 cm among women. .Although the correlation of BMI and WC with CVD risk factors had been well established in many previous studies [32–34] it is not yet well understood how BMI and WC are related to outcomes such as CVD events, all-cause mortality, and CVD-related mortality. Studies have demonstrated that in certain groups of people, such as patients with chronic diseases and those with established coronary artery diseases, a U-shaped correlation exists between BMI and mortality meaning that overweight and mildly obese individuals have lower mortality while patients with either low BMI or too high BMI are more likely to experience CVD events and mortality [35–38] a phenomenon called the obesity paradox [39]. In a study by Adegbija et al. [36], the risk of all-cause mortality decreased by 9% with each standard deviation increase in BMI while boosting by 17% with each standard deviation increase in WC. In another cohort study on Spanish elderly people [38], individuals in the upper quartile of BMI had a 15% lower mortality rate than their counterparts in the lower quartile. After adjustment for WC, the inverse correlation between BMI and mortality became stronger, evidenced by a 37% lower mortality rate in the upper compared to the lower quartile of BMI.

In contrast, before adjustment for BMI, WC was not associated with mortality while after the adjustment, the mortality rate was observed to be higher in the upper WC quartile than in the lower quartile. Also, the same results were observed in a study investigating mortality in patients’ suffering from myocardial infarction [37] reporting hazard ratios of 0.64 and 1.55 for BMI and WC, respectively, per each increase in the standard deviation of mortality. As observed in previous studies, WC seems to be a better predictor of CVD events or mortality than BMI.

In our study, the incidence of CVD events increased from normal weight to overweight, and from overweight to obesity group, in both men (CVD incidence rate of 0.31, 0.43, 0.45 per 10,000 in normal weight, overweight and obese men, respectively) and women (CVD incidence rate of 0.11, 0.21, 0.26 per 10,000 in normal weight, overweight and obese women, respectively). The incidence rate of all-cause mortality was the highest in men with BMI < 25 (0.27 per 10,000). As well, all-cause mortality in obese men was higher than in overweight peers (0.22 vs. 0.21 per 10,000). Among women, the highest all-cause mortality was seen in obese people (0.14 per 10,000), and it was higher in those with BMI < 25 (0.12 per 10,000) compare to women with 25 < BMI < 30 (0.11 per 10,000).

The combination use of BMI and WC can provide a more accurate predictor of mortality, as seen in previous studies [40, 41]. In a study on more than 23 million Korean people, a linear association was noticed between WC and all-cause mortality in all BMI categories [40]. It is well-established that WC varies considerably within any BMI category and shows a notable correlation with health-related risk factors. In a pooled analysis of 11 studies on 650,000 subjects, mortality positively correlated with WC in each BMI category [11]. However, when adjusted for WC, mortality was lower in subjects with higher BMI [42]. The association of WC with CVD events and CVD-related mortality has also been established in other studies [43–45]. In another prospective cohort study, a higher WC predicted higher nonfatal and fatal CVD incidents [45]. Also, in a cohort study on more than 58,000 elderly subjects, a greater WC was associated with a higher relative risk of CVD mortality in any BMI category [44].

The most common sex-specific WC thresholds recommended by National Institute of Health (NIH), which were originally proposed by Lean and colleagues [18] are 102 and 88 cm for men and women, respectively, corresponding to a BMI of 30 kg/m^2^. In recent years many studies suggested different WC cut-off points to predict the incidence of CVD events and incidence of metabolic syndrome as well as cardio metabolic alterations [19–21]. These cut-off values range from 85 to 95 cm in men and 80 to 90 cm in women of different ethnicities. Few studies have evaluated the role of WC thresholds predicting CVD outcomes [22, 23]. In a study by Talaei et al. [22], the optimal WC cut-off point to anticipate CVD events was reported as 99 cm in men and as 103 cm in women. Another study by Hadaegh et al. (2009) in the framework of TLGS with shorter follow up time utilizing different analysis methods and without considering BMI categories suggested the WC threshold of 94.5 cm as the optimal cut-off for predicting CVD events [23]. The above-mentioned cut-offs were similar to those presented in the current study and higher compared to the thresholds suggested for metabolic syndrome or cardiovascular risk factors.

The cut-off values reported in the present study (i.e., 99 cm for men and 103 cm for women), along with the thresholds reported by Talaei et al. [22] (i.e., 93 and 97 cm for men and women, respectively), delivered a low sensitivity. On the other hand, there is a trade-off between sensitivity and specificity, meaning that in order to reach a higher sensitivity, specificity should be sacrificed and vice versa. The optimal cut-off points in our study were defined based on the maximum level of the Youden index. Generally, when WC is used as a screening tool, sensitivity is of greater importance. In the study of Lee et al. [46], the BMI-specific WC thresholds were reported regarding cardiovascular risk factor prediction for values with at least 80% sensitivity. The suggested thresholds were 80 and 89 cm for normal weight and overweight men and 78 and 94 cm for women, respectively, which except in overweight women, are lower values than our suggested thresholds. In our study, the sensitivity ranged from 31.7 to 100%, and their specificity ranged from 38.2 to 78.2%. The lowest sensitivity values for CVD-related mortality (32.6%) and all-cause mortality (31.7%) were observed in men with BMI < 25. In addition, among men with BMI < 25 kg/m^2^, the WC cut-off for all-cause mortality (88 cm) was remarkably higher than that CVD events (82 cm). In this BMI category, WC thresholds of 82 cm (CVD-related mortality) and 80 cm (all-cause mortality) delivered a sensitivity higher than 60%.

According to the results of our study, the WC thresholds obtained for CVD events and all-cause mortality were 82 and 88 cm (normal weight), 95 (overweight) and 103 cm (obese) in men and 82 and 83 cm (normal weight), 89 and 90 cm (overweight) and 99 and 100 cm (obese) in women, respectively. Few studies have evaluated the predictive value of BMI-specific WC cut-off points [24, 47]. In a study by Staiano et al. [47], the WC thresholds reported to best predict cardio metabolic risk factors in the normal weight, overweight, obesity I, and obesity II groups were 82, 95, 107, and 120 cm in men, and 72, 87, 97, and 111 cm in women, respectively. These values are almost the same as those observed in ours study, however, the values obtained for women in the recent study were lower compared to ours. In another study, the WC thresholds predicting a high risk of coronary events in the normal-weight, overweight, obesity I, and obesity II groups were obtained as 82–89, 95–99, 106–110, and 109–125 cm in men ; and 79–81, 90–93, 100–104, and 112–116 cm in women, respectively [24]. Also, these values were close to those observed in our study.

This study has several strengths and limitations. The main strengths of our study include the long median follow-up time, its prospective cohort design, using CVD events and mortality as endpoints, and collection of subjective instead of self-report data. Regarding the limitations of the present study, the data were related to the middle-east Caucasian residents of a metropolitan city in Iran, who cannot be representative of national population. Different methods of WC measurement have been established. In the present study, WC was measured at the umbilical level. Since there are different methods for measuring WC, although it is unlikely for the method of WC measurement to affects the results [13], this point should be considered when comparing the results of different studies.

In conclusion, the results of this study suggested BMI-specific WC thresholds for predicting CVD events, CVD-related and mortality, and all-cause mortality, which can used as a clue for future studies to define more accurate WC cut-off values as a screening tool in different populations. This approach can help better identify individuals who are at a high risk of developing CVD and take effective measures to modify their risk factors.

## Electronic supplementary material

Below is the link to the electronic supplementary material.

Supplementary Material 1

## Data Availability

The datasets used and analyzed during the current study are available from the corresponding author on reasonable request.
