# Supplementary material for: BMI category-specific waist circumference thresholds based on cardiovascular disease outcomes and all-cause mortality: Tehran lipid and glucose study (TLGS)
Source: BMC Public Health. 2023 Jul 5;23:1297. doi: 10.1186/s12889-023-16190-w (PMC10324109; doi:10.1186/s12889-023-16190-w)
Supplement: Supplementary file 1 — Supplementary Material 1 [file 12889_2023_16190_MOESM1_ESM.docx]

**Supplementary Table 1.** Sensitivity and specificity for different waist circumference regarding cardiovascular incident

|  | **Men** | | | | | | | | | **Women** | | | | | | | | |
| --- | --- | --- | --- | --- | --- | --- | --- | --- | --- | --- | --- | --- | --- | --- | --- | --- | --- | --- |
| WC  (cm) | **BMI<25** | | | **25<BMI<30** | | | **BMI>30** | | | **BMI<25** | | | **25<BMI<30** | | | **BMI>30** | | |
|  | sensitivity | specificity | YI | sensitivity | specificity | YI | sensitivity | specificity | YI | sensitivity | specificity | YI | sensitivity | specificity | YI | sensitivity | specificity | YI |
| **80** | 0.783 | 0.343 | 0.126 | 0.999 | 0.012 | 0.011 |  |  |  | 0.618 | 0.579 | 0.197 | 0.972 | 0.107 | 0.079 |  |  |  |
| **81** | 0.747 | 0.398 | 0.145 | 0.986 | 0.015 | 0.001 |  |  |  | 0.577 | 0.649 | 0.226 | 0.936 | 0.145 | 0.081 |  |  |  |
| **82** | 0.709 | 0.454 | 0.163 | 0.986 | 0.019 | 0.005 |  |  |  | 0.577 | 0.681 | 0.258 | 0.927 | 0.169 | 0.096 |  |  |  |
| **83** | 0.639 | 0.509 | 0.148 | 0.987 | 0.024 | 0.011 |  |  |  | 0.542 | 0.714 | 0.256 | 0.926 | 0.201 | 0.127 |  |  |  |
| **84** | 0.595 | 0.562 | 0.157 | 0.981 | 0.034 | 0.015 |  |  |  | 0.425 | 0.758 | 0.183 | 0.899 | 0.246 | 0.145 |  |  |  |
| **85** | 0.533 | 0.616 | 0.149 | 0.982 | 0.05 | 0.032 | 1 | 0.002 | 0.002 | 0.426 | 0.779 | 0.205 | 0.879 | 0.287 | 0.166 | 1 | 0.024 | 0.024 |
| **86** | 0.429 | 0.673 | 0.102 | 0.958 | 0.079 | 0.037 | 1 | 0.002 | 0.002 | 0.353 | 0.813 | 0.166 | 0.86 | 0.337 | 0.197 | 1 | 0.031 | 0.031 |
| **87** | 0.382 | 0.713 | 0.095 | 0.94 | 0.107 | 0.047 | 1 | 0.004 | 0.004 | 0.352 | 0.825 | 0.177 | 0.847 | 0.388 | 0.235 | 1 | 0.044 | 0.044 |
| **88** | 0.345 | 0.74 | 0.085 | 0.912 | 0.143 | 0.055 | 1 | 0.006 | 0.006 | 0.351 | 0.83 | 0.181 | 0.819 | 0.43 | 0.249 | 1 | 0.057 | 0.057 |
| **89** | 0.249 | 0.792 | 0.041 | 0.88 | 0.184 | 0.064 | 1 | 0.007 | 0.007 | 0.27 | 0.854 | 0.124 | 0.791 | 0.476 | 0.267 | 1 | 0.074 | 0.074 |
| **90** | 0.219 | 0.807 | 0.026 | 0.839 | 0.231 | 0.07 | 1 | 0.009 | 0.009 | 0.27 | 0.854 | 0.124 | 0.753 | 0.513 | 0.266 | 0.96 | 0.092 | 0.052 |
| **91** | 0.19 | 0.811 | 0.001 | 0.805 | 0.285 | 0.09 | 1 | 0.011 | 0.011 | 0.193 | 0.876 | 0.069 | 0.661 | 0.575 | 0.236 | 0.951 | 0.123 | 0.074 |
| **92** | 0.169 | 0.808 | -0.023 | 0.74 | 0.342 | 0.082 | 1 | 0.015 | 0.015 | 0.117 | 0.908 | 0.025 | 0.596 | 0.611 | 0.207 | 0.93 | 0.144 | 0.074 |
| **93** | 0.151 | 0.801 | -0.048 | 0.689 | 0.403 | 0.092 | 0.987 | 0.03 | 0.017 | 0.116 | 0.896 | 0.012 | 0.513 | 0.654 | 0.167 | 0.921 | 0.165 | 0.086 |
| **94** | 0.141 | 0.759 | -0.1 | 0.637 | 0.46 | 0.097 | 0.986 | 0.05 | 0.036 | 0.075 | 0.908 | -0.017 | 0.459 | 0.695 | 0.154 | 0.912 | 0.198 | 0.11 |
| **95** | 0.096 | 0.801 | -0.103 | 0.607 | 0.521 | 0.128 | 0.987 | 0.059 | 0.046 | 0.073 | 0.898 | -0.029 | 0.385 | 0.735 | 0.12 | 0.891 | 0.23 | 0.121 |
| **96** |  |  |  | 0.514 | 0.59 | 0.104 | 0.972 | 0.072 | 0.044 |  |  |  | 0.334 | 0.766 | 0.1 | 0.868 | 0.265 | 0.133 |
| **97** |  |  |  | 0.439 | 0.647 | 0.086 | 0.959 | 0.096 | 0.055 |  |  |  | 0.283 | 0.795 | 0.078 | 0.818 | 0.31 | 0.128 |
| **98** |  |  |  | 0.358 | 0.702 | 0.06 | 0.931 | 0.128 | 0.059 |  |  |  | 0.235 | 0.821 | 0.056 | 0.787 | 0.356 | 0.143 |
| **99** |  |  |  | 0.3 | 0.744 | 0.044 | 0.902 | 0.167 | 0.069 |  |  |  | 0.208 | 0.835 | 0.043 | 0.766 | 0.405 | 0.171 |
| **100** |  |  |  | 0.253 | 0.776 | 0.029 | 0.888 | 0.211 | 0.099 |  |  |  | 0.182 | 0.847 | 0.029 | 0.756 | 0.44 | 0.196 |
| **101** |  |  |  |  |  |  | 0.815 | 0.278 | 0.093 |  |  |  |  |  |  | 0.68 | 0.493 | 0.173 |
| **102** |  |  |  |  |  |  | 0.774 | 0.332 | 0.106 |  |  |  |  |  |  | 0.639 | 0.526 | 0.165 |
| **103** |  |  |  |  |  |  | 0.749 | 0.382 | 0.131 |  |  |  |  |  |  | 0.597 | 0.567 | 0.164 |
| **104** |  |  |  |  |  |  | 0.658 | 0.438 | 0.096 |  |  |  |  |  |  | 0.544 | 0.604 | 0.148 |
| **105** |  |  |  |  |  |  | 0.616 | 0.482 | 0.098 |  |  |  |  |  |  | 0.512 | 0.635 | 0.147 |

BMI, body mass index; WC, waist circumference; YI, Youden’s index.

**Supplementary Table 2.** Sensitivity and specificity for different waist circumference regarding all-cause mortality

|  | **Men** | | | | | | | | | **Women** | | | | | | | | |
| --- | --- | --- | --- | --- | --- | --- | --- | --- | --- | --- | --- | --- | --- | --- | --- | --- | --- | --- |
| WC  (cm) | **BMI<25** | | | **25<BMI<30** | | | **BMI>30** | | | **BMI<25** | | | **25<BMI<30** | | | **BMI>30** | | |
|  | sensitivity | specificity | YI | sensitivity | specificity | YI | sensitivity | specificity | YI | sensitivity | specificity | YI | sensitivity | specificity | YI | sensitivity | specificity | YI |
| **80** | 0.657 | 0.35 | 0.007 | 0.987 | 0.012 | -0.001 |  |  |  | 0.656 | 0.576 | 0.232 | 0.979 | 0.108 | 0.087 |  |  |  |
| **81** | 0.589 | 0.409 | -0.002 | 0.987 | 0.015 | 0.002 |  |  |  | 0.584 | 0.647 | 0.231 | 0.981 | 0.146 | 0.127 |  |  |  |
| **82** | 0.566 | 0.468 | 0.034 | 0.987 | 0.019 | 0.006 |  |  |  | 0.55 | 0.681 | 0.231 | 0.958 | 0.17 | 0.128 |  |  |  |
| **83** | 0.501 | 0.525 | 0.026 | 0.987 | 0.024 | 0.011 |  |  |  | 0.551 | 0.71 | 0.261 | 0.958 | 0.202 | 0.16 |  |  |  |
| **84** | 0.469 | 0.582 | 0.051 | 0.987 | 0.034 | 0.021 |  |  |  | 0.448 | 0.753 | 0.201 | 0.958 | 0.248 | 0.206 |  |  |  |
| **85** | 0.404 | 0.644 | 0.048 | 0.963 | 0.051 | 0.014 | 1 | 0 | 0 | 0.415 | 0.777 | 0.192 | 0.957 | 0.29 | 0.247 | 1 | 0.024 | 0.024 |
| **86** | 0.351 | 0.696 | 0.047 | 0.927 | 0.08 | 0.007 | 1 | 0.002 | 0.002 | 0.349 | 0.81 | 0.159 | 0.956 | 0.341 | 0.297 | 1 | 0.031 | 0.031 |
| **87** | 0.339 | 0.734 | 0.073 | 0.88 | 0.108 | -0.012 | 1 | 0.004 | 0.004 | 0.278 | 0.837 | 0.115 | 0.932 | 0.393 | 0.325 | 1 | 0.044 | 0.044 |
| **88** | 0.317 | 0.761 | 0.078 | 0.857 | 0.144 | 0.001 | 1 | 0.006 | 0.006 | 0.276 | 0.845 | 0.121 | 0.909 | 0.437 | 0.346 | 1 | 0.057 | 0.057 |
| **89** | 0.25 | 0.803 | 0.053 | 0.845 | 0.186 | 0.031 | 1 | 0.007 | 0.007 | 0.239 | 0.859 | 0.098 | 0.885 | 0.486 | 0.371 | 1 | 0.075 | 0.075 |
| **90** | 0.195 | 0.835 | 0.03 | 0.798 | 0.233 | 0.031 | 1 | 0.009 | 0.009 | 0.239 | 0.859 | 0.098 | 0.863 | 0.523 | 0.386 | 1 | 0.092 | 0.092 |
| **91** | 0.172 | 0.844 | 0.016 | 0.775 | 0.289 | 0.064 | 1 | 0.011 | 0.011 | 0.242 | 0.843 | 0.085 | 0.759 | 0.588 | 0.347 | 1 | 0.124 | 0.124 |
| **92** | 0.141 | 0.86 | 0.001 | 0.678 | 0.348 | 0.026 | 1 | 0.015 | 0.015 | 0.172 | 0.863 | 0.035 | 0.759 | 0.619 | 0.378 | 1 | 0.144 | 0.144 |
| **93** | 0.12 | 0.867 | -0.013 | 0.631 | 0.411 | 0.042 | 1 | 0.03 | 0.03 | 0.14 | 0.866 | 0.006 | 0.718 | 0.658 | 0.376 | 1 | 0.166 | 0.166 |
| **94** | 0.119 | 0.834 | -0.047 | 0.582 | 0.47 | 0.052 | 0.926 | 0.05 | -0.024 | 0.105 | 0.858 | -0.037 | 0.677 | 0.698 | 0.375 | 1 | 0.198 | 0.198 |
| **95** | 0.087 | 0.856 | -0.057 | 0.534 | 0.54 | 0.074 | 0.926 | 0.06 | -0.014 | 0.066 | 0.901 | -0.033 | 0.591 | 0.737 | 0.328 | 1 | 0.23 | 0.23 |
| **96** |  |  |  | 0.427 | 0.614 | 0.041 | 0.925 | 0.073 | -0.002 |  |  |  | 0.502 | 0.77 | 0.272 | 0.981 | 0.267 | 0.248 |
| **97** |  |  |  | 0.379 | 0.674 | 0.053 | 0.926 | 0.097 | 0.023 |  |  |  | 0.434 | 0.798 | 0.232 | 0.957 | 0.311 | 0.268 |
| **98** |  |  |  | 0.284 | 0.734 | 0.018 | 0.928 | 0.13 | 0.058 |  |  |  | 0.348 | 0.826 | 0.174 | 0.931 | 0.357 | 0.288 |
| **99** |  |  |  | 0.249 | 0.778 | 0.027 | 0.852 | 0.169 | 0.021 |  |  |  | 0.326 | 0.838 | 0.164 | 0.932 | 0.407 | 0.339 |
| **100** |  |  |  | 0.213 | 0.817 | 0.03 | 0.852 | 0.215 | 0.067 |  |  |  | 0.328 | 0.841 | 0.169 | 0.88 | 0.444 | 0.324 |
| **101** |  |  |  |  |  |  | 0.776 | 0.283 | 0.059 |  |  |  |  |  |  | 0.826 | 0.498 | 0.324 |
| **102** |  |  |  |  |  |  | 0.777 | 0.339 | 0.116 |  |  |  |  |  |  | 0.801 | 0.53 | 0.331 |
| **103** |  |  |  |  |  |  | 0.78 | 0.391 | 0.171 |  |  |  |  |  |  | 0.754 | 0.572 | 0.326 |
| **104** |  |  |  |  |  |  | 0.593 | 0.451 | 0.044 |  |  |  |  |  |  | 0.727 | 0.607 | 0.334 |
| **105** |  |  |  |  |  |  | 0.555 | 0.499 | 0.054 |  |  |  |  |  |  | 0.675 | 0.641 | 0.316 |

BMI, body mass index; WC, waist circumference; YI, Youden’s index.
